# Antibody responses to flagellin C and *Streptococcus gallolyticus* pilus proteins in colorectal cancer

**DOI:** 10.1038/s41598-019-47347-6

**Published:** 2019-07-26

**Authors:** Julia Butt, Nerea Fernández de Larrea, Harold Tjalsma, Rian Roelofs, Ikuko Kato, Vicente Martín, Beatriz Pérez-Gómez, Victor Moreno, Trinidad Dierssen-Sotos, Jesús Castilla, Guillermo Fernández-Tardón, Pilar Amiano, Dolores Salas, Juan Alguacil, José Juan Jiménez-Moleón, José María Huerta, Silvia de Sanjosé, Rosa del Campo, Manolis Kogevinas, Marina Pollán, Michael Pawlita, Tim Waterboer, Annemarie Boleij, Nuria Aragonés

**Affiliations:** 1 Infection and Cancer Epidemiology Group, German Cancer Research Center (DKFZ), Heidelberg, Germany; 2 Faculty of Biosciences, University of Heidelberg, Heidelberg, Germany; 3 Cancer and Environmental Epidemiology Unit, National Center for Epidemiology, Carlos III Institute of Health, Madrid, Spain; 4 Consortium for Biomedical Research in Epidemiology and Public Health (CIBERESP), Madrid, Spain; 5 Independent researcher. Experimental work conducted at the Department of Laboratory Medicine, Radboud University Medical Centre (RadboudUMC), Nijmegen, The Netherlands; 6 Department of Laboratory Medicine, Radboud Institute for Molecular Life Sciences (RIMLS), Radboud university medical centre (Radboudumc), Nijmegen, The Netherlands; 7 Departments of Oncology and Pathology, Wayne State University, Detroit, Michigan USA; 8 The Research Group in Gene Environment and Health Interactions, University of León, León, Spain; 9 Cardiovascular and Metabolic Diseases Unit, National Center for Epidemiology, Carlos III Institute of Health, Madrid, Spain; 10 Oncology Data Analytics Program, Catalan Institute of Oncology (ICO), Hospitalet de Llobregat, Barcelona, Spain; 11 Colorectal Cancer Group, ONCOBELL Program, Bellvitge Biomedical Research Institute (IDIBELL), Hospitalet de Llobregat, Barcelona, Spain; 12 Department of Clinical Sciences, Faculty of Medicine, University of Barcelona, Barcelona, Spain; 13 University of Cantabria – IDIVAL, Santander, Spain; 14 Public Health Institute of Navarra, IdiSNA- Navarra Institute for Health Research, Pamplona, Spain; 15 IUOPA, University of Oviedo, Oviedo, Spain; 16 Public Health Division of Gipuzkoa, BioDonostia Research institute, San Sebastian, Spain; 17 Cancer and Public Health Area, FISABIO – Public Health, Valencia, Spain; 18 General Directorate Public Health, Valencian Community, Valencia, Spain; 19 Natural Resources, Health and Environment Research Center (RENSMA), University of Huelva, Huelva, Spain; 20 Granada Health Research Institute (ibs.GRANADA), Granada, Spain; 21 Department of Preventive Medicine and Public Health, University of Granada, Granada, Spain; 22 Department of Epidemiology, Murcia Regional Health Council, IMIB-Arrixaca, Murcia, Spain; 23 Cancer Epidemiology Research Program, Catalan Institute of Oncology-IDIBELL, L’Hospitalet de Llobregat, Barcelona, Spain; 24 PATH, Reproductive Health, Seattle, USA; 25 Department of Microbiology, Ramón y Cajal Health Research Institute (IRYCIS), Hospital Universitario Ramón y Cajal, Madrid, Spain; 26 Spanish Network for Research in Infectious Diseases (REIPI), Madrid, Spain; 27 ISGlobal, Barcelona, Spain; 28 IMIM (Hospital del Mar Medical Research Institute), Barcelona, Spain; 29 Pompeu Fabra University (UPF), Barcelona, Spain; 30 Department of Pathology, Radboud Institute for Molecular Life Sciences (RIMLS), Radboud university medical centre (Radboudumc), Nijmegen, The Netherlands; 31 Epidemiology Section, Public Health Division, Department of Health of Madrid, Madrid, Spain

**Keywords:** Cancer epidemiology, Diagnostic markers

## Abstract

Antibodies to *Streptococcus gallolyticus* subspecies *gallolyticus* (SGG) have been associated with colorectal cancer (CRC). Because SGG may correlate with impaired gut epithelia, we assessed the association of antibodies to bacterial flagellin C (FliC), a measure potentially related to this impairment, with CRC and the CRC-specific interaction with antibodies to SGG proteins. Antibodies to FliC and SGG pilus proteins Gallo2178 and Gallo2179 were measured in two independent studies, a combined study from Nijmegen and Detroit (93 CRC cases, 74 controls) and a replication data set including 576 cases and 576 controls from the Spanish multicenter multicase-control study (MCC-Spain). Logistic regression was applied to assess whether antibodies to FliC were associated with CRC and modified the association of antibodies to SGG proteins with CRC. Antibodies to FliC were associated with those to SGG Gallo2178 among CRC cases, resulting in an interaction in the association of antibodies to Gallo2178 with CRC (p = 0.007). This association was only present among individuals with high antibody responses to FliC (OR: 2.42, 95% CI: 1.45–4.06). In conclusion, our findings suggest that colorectal tumorigenesis could be accompanied by an impaired integrity of the epithelium that could result in associated increased antibody responses to bacterial proteins.

## Introduction

Colorectal cancer (CRC) is the fourth most common cancer worldwide with in total 1.36 million newly diagnosed cases in 2012^1^. Risk factors include older age, male sex, certain dietary habits, tobacco and alcohol consumption, obesity, family history of CRC as well as history of inflammatory bowel diseases^2^. Interest in a potential association of bacteria residing in the gut with CRC development has increased during the past years^3,4^. In particular, *Streptococcus gallolyticus* subspecies *gallolyticus* (SGG) was shown to be associated with CRC in several epidemiological studies but also in some mechanistic studies^5–14^. Boleij *et al*. showed that antibody responses to SGG pilus proteins Gallo2178 and Gallo2179, both being hypothesized to be involved in virulence of the bacterium^15,16^, were selectively detectable in adenoma and CRC cases in two studies from Nijmegen (Netherlands), and Detroit (USA)^6^. We reproduced this finding in two case-control studies, including a subset of the multicentric multicase-control study MCC-Spain, as well as one prospective study and demonstrated a significant association of antibody responses to Gallo2178 and Gallo2179 with an approximately 2-fold increased risk of developing CRC^8,9,12^.

SGG is a rare gut microbe in humans and occasionally causes systemic infection, facilitated by increased mucosal permeability, as seen in tumor development, as well as by strain specific virulence^4^. An impaired gut barrier, however, should also enable other organisms to invade the otherwise well-protected gut epithelium. Bacterial products like lipopolysaccharide (LPS), also called endotoxin, or motility-proteins like flagellin C (FliC) are wide-spread among bacterial species and upon infection are targets of the host immune system^17^. Several studies assessed the association of these common bacterial products with diseases of the gut, e.g. Crohn’s disease or CRC and its precursors^7,18–21^. Kang *et al*. found that individuals with high plasma endotoxin levels had a 1.4-fold increased risk of having adenomas^20^, and Kong *et al*. found that high levels of antibody responses to *Escherichia coli* (*E*. *coli*) LPS and FliC were prospectively associated with CRC in men (OR: 1.66, 95% CI: 1.10–2.51)^19^. In two other studies from the Netherlands and USA, *Salmonella* FliC was associated with colorectal adenomas (OR 4.71, 95% CI 1.10–20.14) and carcinomas (OR 3.09, 95% CI 1.22–7.79), while endotoxin levels were not^7,21^.

Using serological data obtained in the CRC and adenoma case-control studies from Nijmegen and Detroit and data obtained in MCC-Spain as a replication study, we here assessed whether antibody responses to FliC were simultaneously associated with CRC and with antibody responses to Gallo2178 and Gallo2179, as suggested by the gut permeability hypothesis.

## Results

### Association of antibody responses to FliC with CRC

In the Nijmegen/Detroit study, the OR for adenoma/CRC significantly increased with higher FliC quartile (p_trend_ = 0.011) with a statistically significant association of the fourth *versus* the first quartile (OR: 2.32, 95% CI: 1.01–5.33) (Table 1, see Supplementary Table S2 for individual studies). In the replication study MCC-Spain, the OR for CRC did not show a positive trend, although the highest risk was found in the highest FliC quartile (Q4 vs Q1, OR: 1.14, 95% CI: 0.81–1.59).

**Table 1:** Association of antibody response to FliC with adenoma/CRC in the Nijmegen/Detroit and MCC-Spain studies.

Study	FliC	Total n (%)	Controls n (%)	Cases^a^ n (%)	OR (95%CI)^b^	p_trend_
Nijmegen/Detroit studies	Q1	38 (23)	19 (26)	19 (20)	ref	
	Q2	29 (17)	19 (26)	10 (11)	0.53 (0.20–1.43)	
	Q3	37 (22)	17 (23)	20 (22)	1.18 (0.48–2.91)	
	Q4	63 (38)	19 (26)	44 (47)	**2.32 (1.01–5.33)**	**0.011**
MCC-Spain	Q1	296 (26)	145 (25)	151 (26)	ref	
	Q2	254 (22)	143 (25)	111 (19)	0.75 (0.53–1.06)	
	Q3	288 (25)	144 (25)	144 (25)	0.99 (0.70–1.38)	
	Q4	314 (27)	144 (25)	170 (30)	1.14 (0.81–1.59)	0.247

^a^Cases include n = 23 adenoma and n = 70 CRC cases for Nijmegen/Detroit studies, all cases were CRC in MCC Spain; ^b^Logistic regression model, Nijmegen/Detroit study: no further adjustment, MCC-Spain: adjustment for age, sex, province, education, BMI, smoking and family history of CRC; significant associations (p-value < 0.05) are marked in bold font; Q = quartile.

### Association of antibody responses to FliC with antibody positivity to SGG pilus proteins Gallo2178 and Gallo2179

We assessed in both studies, Nijmegen/Detroit and MCC-Spain, whether positivity to SGG pilus proteins was associated with antibody responses to FliC. In the total population of the Nijmegen/Detroit study, there was an association between antibody response to FliC and positivity to Gallo2179 (p = 0.040) but not to Gallo2178 (p = 0.072) (Table 2). However, when analyzing cases and controls separately we observed that antibody positivity to Gallo2178 and Gallo2179 were significantly associated with FliC antibody response among cases (p = 0.023 and p = 0.021, respectively) but not among controls (p = 0.658 and p = 0.471, respectively) (see Supplementary Table S3 for individual studies).

**Table 2:** Antibody response to FliC and association with SGG Gallo2178 and Gallo2179 antibody positivity.

FliC quartile	Gallo2178		p-value^b^	Gallo2179		p-value^b^
	neg n (%)	pos n (%)		neg n (%)	pos n (%)	
**Nijmegen/Detroit studies**						
**Total**	**150 (100)**	**17 (100)**		**151 (100)**	**16 (100)**	
Q1	36 (24)	2 (12)		35 (23)	3 (19)	
Q2	27 (18)	2 (12)		28 (19)	1 (6)	
Q3	29 (19)	8 (47)		29 (19)	8 (50)	
Q4	58 (39)	5 (29)	0.072	59 (39)	4 (25)	**0.040**
**Controls**	**65 (100)**	**9 (100)**		**66 (100)**	**8 (100)**	
Q1	17 (26)	2 (22)		16 (24)	3 (38)	
Q2	18 (28)	1 (11)		18 (27)	1 (13)	
Q3	14 (22)	3 (33)		14 (21)	3 (38)	
Q4	16 (25)	3 (33)	0.658	18 (27)	1 (13)	0.471
**Cases** ^**a**^	**85 (100)**	**8 (100)**		**85 (100)**	**8 (100)**	
Q1	19 (22)	0 (0)		19 (22)	0 (0)	
Q2	9 (11)	1 (13)		10 (12)	0 (0)	
Q3	15 (18)	5 (63)		15 (18)	5 (63)	
Q4	42 (49)	2 (25)	**0.023**	41 (48)	3 (37)	**0.021**
**MCC-Spain**						
**Total**	**1012 (100)**	**140 (100)**		**1015 (100)**	**137 (100)**	
Q1	268 (26)	28 (20)		269 (27)	27 (20)	
Q2	226 (22)	28 (20)		225 (22)	29 (21)	
Q3	247 (24)	41 (29)		250 (25)	38 (28)	
Q4	271 (27)	43 (31)	0.242	271 (27)	43 (31)	0.300
**Controls**	**519 (100)**	**57 (100)**		**520 (100)**	**56 (100)**	
Q1	130 (25)	15 (26)		135 (26)	10 (18)	
Q2	126 (24)	17 (30)		127 (24)	16 (29)	
Q3	132 (25)	12 (21)		126 (24)	18 (32)	
Q4	131 (25)	13 (23)	0.757	132 (25)	12 (21)	0.354
**Cases** ^**a**^	**493 (100)**	**83 (100)**		**495 (100)**	**81 (100)**	
Q1	138 (28)	13 (16)		134 (27)	17 (21)	
Q2	100 (20)	11 (13)		98 (20)	13 (16)	
Q3	115 (23)	29 (35)		124 (25)	20 (25)	
Q4	140 (28)	30 (36)	**0.011**	139 (28)	31 (38)	0.268

^a^Cases include n = 23 adenoma and n = 70 CRC cases for Nijmegen/Detroit studies, all cases were CRC in MCC Spain; ^b^Chi-Square test; significant associations (p-value < 0.05) are marked in bold font.

In the replication study MCC-Spain, we partly reproduced the findings observed in the Nijmegen/Detroit study (Table 2): While there was no association between FliC quartile and positivity to neither Gallo2178 nor to Gallo2179 in the whole population, antibody positivity to Gallo2178 was significantly associated with FliC antibody response among cases (p = 0.011). The majority of cases positive for Gallo2178 (88% in the Nijmegen/Detroit study and 71% in MCC-Spain) had simultaneously antibody responses to FliC in the upper quartiles 3 and 4 (>379.5 MFI).

### Association of antibody responses to SGG Gallo2178 and Gallo2179 with CRC stratified by FliC antibody response in MCC-Spain

The abovementioned analysis revealed a significant association between antibody positivity to SGG proteins with high antibody responses to FliC only among CRC cases. We performed an analysis of the association between antibody responses to SGG pilus proteins and CRC stratified by median MFI to FliC. The analysis was performed in the MCC-Spain study only since the sample size of the Nijmegen/Detroit study did not allow analysis with sufficient statistical power.

Among individuals with antibody responses to FliC below the median MFI, the association between antibody responses to SGG pilus proteins and CRC was absent (Gallo2178: OR: 0.85, 95%CI: 0.48–1.49; Gallo2179: OR: 1.26, 95% CI: 0.72–2.20) (Table 3). However, among individuals with an antibody response to FliC above the median MFI, the OR for CRC with antibody positivity to Gallo2178 and Gallo2179 was increased and reached statistical significance for Gallo2178 (Gallo2178: OR: 2.42, 95%CI: 1.45–4.06; Gallo2179: OR: 1.63, 95% CI: 0.99–2.68). This interaction was significant for Gallo2178 (p_interaction_ = 0.007).

**Table 3:** Association of SGG pilus proteins Gallo2178 and Gallo2179 with CRC, overall and stratified by FliC median antibody response in MCC-Spain.

	Antigen		Controls n (%)	Cases n (%)	OR (95%CI)^a^	p-value^a^	p_interaction_
Overall	Gallo2178	Neg	519 (90)	493 (86)			
		Pos	57 (10)	83 (14)	**1.57 (1.09–2.27)**	**0.016**	
FliC Q1/Q2	Gallo2178	Neg	256 (89)	238 (91)			
		Pos	32 (11)	24 (9)	0.85 (0.48–1.49)	0.564	
FliC Q3/Q4	Gallo2178	Neg	263 (91)	255 (81)			
		Pos	25 (9)	59 (19)	**2.42 (1.45–4.06)**	**0.0007**	**0.007**
Overall	Gallo2179	Neg	520 (90)	495 (86)			
		Pos	56 (10)	81 (14)	**1.46 (1.01–2.11)**	**0.045**	
FliC Q1/Q2	Gallo2179	Neg	262 (91)	232 (89)			
		Pos	26 (9)	30 (11)	1.26 (0.72–2.20)	0.427	
FliC Q3/Q4	Gallo2179	Neg	258 (90)	263 (84)			
		Pos	30 (10)	51 (16)	1.63 (0.99–2.68)	0.056	0.499

^a^Logistic regression model with adjustment for age, sex, province, education, BMI, smoking and family history of CRC; significant associations (p-value < 0.05) are marked in bold font.

## Discussion

In this study we assessed the association of antibody responses to FliC with CRC as well as a potential interaction with antibody responses to SGG pilus proteins Gallo2178 and Gallo2179. We found a significant trend for higher antibody responses to FliC in CRC cases compared to controls in the Nijmegen/Detroit study, which could not be reproduced in the replication study MCC-Spain. However, antibody responses to FliC were associated with antibody responses to SGG pilus protein Gallo2178 in CRC cases of both studies resulting in a significantly stronger association of CRC with antibody positivity to Gallo2178 among individuals with high antibody responses to FliC compared to those exhibiting low responses to FliC.

FliC is a motility protein conserved among several gram-negative bacteria including *Salmonella* and *Escherichia coli*. It is recognized by the host immune system, specifically by TLR5, when it crosses the gut epithelium as it might be given in the case of a disturbed gut epithelial integrity as a result of a developing tumor^22^. We hypothesize that increased gut permeability in CRC development is related to an increased immune response to certain gut bacteria and that therefore antibody responses to FliC and CRC-related SGG Gallo2178 and Gallo2179 will be increased and correlated in CRC cases in contrast to controls. Our results support this hypothesis since antibody responses to SGG Gallo2178 were specifically associated with CRC in the presence of those to FliC, suggesting increased antigen sampling by the immune system. In this respect, the association of CRC with antibodies to bacterial proteins could be attributed to a complex interplay of several bacteria rather than to a single bacterial species, either invading colorectal tissue simultaneously or consecutively dependent on tumor development as proposed in the bacteria-driver passenger model^4^. Certain bacteria might simply be bystanders without influence on CRC development, while others might contribute to progression, e.g. by induction of inflammation.

SGG has previously been shown to promote tumor growth, *in vitro* using cell lines but also *in vivo* in mouse models. SGG may benefit from cancer metabolites in the tumor environment, and may shape its own niche environment in the developing tumor^11,13–15^. The effects on cell proliferation are correlated with adherence abilities of the respective SGG strains to the epithelium and can depend on the encoded pili, i.e. those expressed from loci *pil1* (Gallo2178 and Gallo2179), *pil2* and *pil3*^16^. Furthermore, certain strain dependencies within SGG might relate to unidentified virulence factors besides *pil1–3* to adhere to and colonize colon epithelia of CRC patients^13^. Comparative genomics of eight SGG strains from human blood and feces recently revealed that complete *pil1-3* loci are only present in SGG causing bacteremia and/or endocarditis, while the fecal SGG may carry truncated versions of the *pil1-3* loci^23,24^. This could also explain the fact that not every patient with a high FliC-titer also expresses measurable antibody responses towards both, Gallo2178 and Gallo2179.

A major strength of this work is the analysis of two separate studies, the Nijmegen/Detroit study as well as MCC-Spain as a replication study, which allowed us to assess reproducibility of the results in studies that were independently conducted. Their differences in study design and serological methodology applied, however, could also have led to the observed association of antibody responses to FliC with adenoma/CRC being present in the Nijmegen/Detroit study only. The Nijmegen/Detroit study included adenoma patients and stage I and II CRC cases whereas MCC-Spain comprised only CRC cases, including all stages. We performed an analysis separately by stage (stage I/II versus stage III/IV) in MCC-Spain and found no difference in the association of antibody responses to FliC with CRC by stage (data not shown). However, it still remains to be elucidated whether these bacteria have a role primarily in adenoma development. Furthermore, as described above, different SGG strains exhibit a distinct virulence potential by differential expression of pilus loci. In addition, the natural history of antibody responses raised against different proteins of SGG is unknown. Longitudinal studies are needed to give further insight when in relation to CRC development sero-conversion to the respective bacterial proteins appears and whether this could even differ in time for proteins of the same pilus loci. The effect on the reported associations of using ELISA in the Nijmegen/Detroit study as opposed to multiplex serology in MCC-Spain cannot be determined from the given data since direct comparisons with both assays in the same samples were not available. We therefore cannot exclude that the applied serological method further may have affected the observed differences in results between studies.

A further limitation of this study was the design and small sample size of the Nijmegen/Detroit study. We combined two independent small studies, one from Nijmegen and one from Detroit (Supplementary Tables S2 and S3). Additionally, adjusting for potential confounders was only possible in the replication study MCC-Spain. The low number of sero-positives (e.g. n = 83 Gallo2178 and n = 81 Gallo2179 sero-positive cases in MCC-Spain) further refrained us from analyzing the antibody responses to Gallo2178 and Gallo2179 in quartiles in relation to FliC quartiles. This analysis of a correlation between antibody level to FliC and Gallo2189 and Gallo2179 would further strengthen the reported results but needs larger datasets for sufficient statistical power. In addition, reverse causality cannot be excluded in a case-control study and the results presented here need to be verified in prospective studies.

In conclusion, we demonstrated that antibody responses to SGG pilus protein Gallo2178 were associated with antibody responses to FliC specifically in CRC cases. This finding suggests that CRC development may be associated with increased gut permeability and concomitant antibody responses to specific CRC-associated bacteria, like SGG.

## Methods

### Study population

#### Nijmegen and detroit studies

The study populations have been described previously^6,7,21^. Serum samples from 37 stage I/II CRC and 12 adenoma patients, who had been admitted to the Radboud University Medical Center (Nijmegen, the Netherlands) were collected. Serum from 27 healthy blood donors >50 years of age were used as controls. Plasma samples from patients who participated in a population-based case-control study in Metropolitan Detroit (USA) were included in the study comprising 26 stage I/II CRC patients, 7 CRC patients with unknown stage, 11 adenoma patients and 47 healthy controls. The use of the samples was approved by the local medical ethical committees (CMO Nijmegen/Arnhem #2006/078 and Wayne State University Human Investigation Committee #0409000504). Serum and plasma samples were stored at −80 °C until use.

#### MCC-Spain study

MCC-Spain is a multicentric multicase-control study assessing the impact of environmental exposures and their interaction with genetic factors on five cancers in Spain^8,25^. Individuals with histologically confirmed malignant disease were recruited between 2008 and 2013 in hospitals of 12 Spanish provinces. Controls free of these cancers were randomly sampled from general practitioner’s lists at primary health centers located in the catchment areas of the hospitals where cases were recruited and frequency-matched to cases by age, sex and province. Participants completed a questionnaire through personal interview and donated a blood sample. For the present study, 576 CRC cases and 576 controls were analyzed. Population characteristics are shown in Supplementary Table S1.

The protocol of MCC-Spain was approved by the Clinical Research Ethic Committee of the Clínico San Carlos Hospital, the Clinical Research Ethic Committee of the Ramón y Cajal Hospital, the Clinical Research Ethic Committee of the Municipal Healthcare Institute of Barcelona, the Clinical Research Ethic Committee of Navarra, and the Research and Animal Well-being Ethic Committee of the Carlos III Health Institute. All the procedures contributing to this work comply with the ethical standards of the relevant national and institutional committees on human experimentation and with the Helsinki Declaration of 1975, as revised in 2008. All participants signed an informed consent.

### Gallo2178, Gallo2179 and FliC ELISA

The ELISA assays for Gallo2178, Gallo2179 and FliC have been described previously^6,7,21^. In short, ELISA-plates were coated with FliC (InvivoGen, San Diego, CA, USA), or nickel-affinity purified recombinant expressed proteins Gallo2178 or Gallo2179 (antigen, Ag)^6^. For each antigen-coated well, a second well on the same plate was incubated with coating buffer without antigen (*blank*). Serum or plasma samples were added to the wells and incubated. Bound serum antibodies were detected using horseradish peroxidase (HRP)-labeled goat anti-human IgG (1:25.000; Jackson Immunoresearch). Optical density of the samples was measured in duplicate and titers were calculated as the mean OD450_*Ag*_ − OD450_*blank*_ and expressed as arbitrary FliC, Gallo2178 or Gallo2179 units based on reference samples from *Salmonella typhimurium*- (*S*. *typhimurium)*, or *S*. *gallolyticus*-infected patients, respectively. Cut-off values for positive Gallo2179 and Gallo2178 response were set as published before with 78% specificity (Gallo2179 > 12.4 (Detroit), Gallo2179 > 9.2 (Nijmegen), Gallo2178 > 2.0 (Detroit) and Gallo2178 > 3.7 (Nijmegen)). Quartiles for FliC were calculated based on the FliC titers of the control group and set at Q1: <2.14, Q2: 2.14 −< 3.18, Q3: 3.18 −< 6.63, and Q4: ≥6.63 (Detroit) and Q1: <1.16, Q2: 1.16 −< 1.70, Q3: 1.70 −< 3.00, and Q4: ≥3.00 (Nijmegen).

### Gallo2178, Gallo2179 and FliC multiplex serology

MCC-Spain serum samples were sent on dry ice to the German Cancer Research Center (DKFZ, Heidelberg, Germany) and analyzed in a final 1:500 dilution. Multiplex serology was performed as described previously^8,26^. His_6_-tagged recombinantly expressed and affinity-purified SGG pilus proteins Gallo2178 and Gallo2179^6^ as well as *S*. *typhimurium* FliC (InvivoGen, San Diego, CA, USA) were directly cross-linked onto fluorescence-labeled polystyrene beads (SeroMap, Luminex Corp., Austin, TX, USA). Mixing of the different antigen-coupled bead sets allowed analysis of antibody responses in a serum against several antigens in one reaction. A Luminex analyser (Luminex Corp., Austin, TX, USA) distinguishes the bead sets and simultaneously quantifies the amount of bound serum antibody by a secondary antibody conjugated with a fluorescent reporter (Streptavidin-R-Phycoerythrin). The level of antibody response was given as median fluorescence intensity (MFI) of at least 100 beads per set measured.

Cut-off definition for antibody responses to SGG Gallo2178 and Gallo2179 was arbitrary as previously described^8^ and defined 10% of controls as positive (cut-off: Gallo2178: 21 MFI; Gallo2179: 506 MFI). Antibody responses to FliC were analyzed in quartiles as defined in controls (Q1: ≤159 MFI; Q2: 159.5–379.5 MFI; Q3: 380–938.5 MFI; Q4: >938.5 MFI).

### Statistical analysis

Nijmegen and Detroit studies were analyzed as a combined study in the main analysis to increase statistical power.

Associations between antibody responses to FliC, and Gallo2178 and Gallo2179 in the Nijmegen/Detroit study and in the MCC-Spain study were analyzed using a Chi-square test.

Associations of antibody responses to FliC, Gallo2178 and Gallo2179 with CRC were assessed using logistic regression models to compute odds ratios (OR) and 95% confidence intervals (95% CI). A p-value of <0.05 was considered statistically significant. For the Nijmegen/Detroit studies, additional sociodemographic information was available only for part of the samples and thus no further adjustment was performed. For the replication in the MCC-Spain study, multivariable logistic regression models were applied with adjustment for age, sex, province, education (low (primary school or lower), intermediate (secondary school), high (University)), BMI (kg/m^2^, <25, 25 −< 30, ≥30), smoking (never, former, current) and family history of CRC in first or second-degree relatives. In the MCC-Spain study, we further performed a stratified analysis by median FliC MFI (379.5 MFI) to address whether antibody responses to FliC affected the association of antibody positivity to SGG pilus proteins with CRC. We added an interaction term between FliC (below/above median MFI) and Gallo2178 or Gallo2179 antibody positivity and performed a likelihood ratio test to assess statistical significance.

## Supplementary information

Supplementary tables S1, S2, S3

Dataset 1

## Data Availability

The authors confirm that the study data of the Nijmegen/Detroit studies are available as supplementary material. The study data of MCC-Spain is available only upon reasonable request to Nerea Fernández de Larrea (nfernandez@isciii.es) due to personal data safety protection regulations.
