# Supplementary material for: Antibody responses to flagellin C and Streptococcus gallolyticus pilus proteins in colorectal cancer
Source: Sci Rep. 2019 Jul 26;9:10847. doi: 10.1038/s41598-019-47347-6 (PMC6659640; doi:10.1038/s41598-019-47347-6)
Supplement: Supplementary file 1 — Supplementary tables S1, S2, S3 [file 41598_2019_47347_MOESM1_ESM.pdf]

Supplementary tables S1, S2, and S3

Manuscript title: Antibody responses to flagellin C and *Streptococcus gallolyticus* pilus proteins in colorectal cancer

Authors
Julia Butt, Nerea Fernández de Larrea, Harold Tjalsma, Rian Roelofs, Ikuko Kato, Vicente Martín, Beatriz Pérez-Gómez, Victor Moreno, Trinidad Dierssen-Sotos, Jesús Castilla, Guillermo Fernández-Tardón, Pilar Amiano, Dolores Salas, Juan Alguacil, José Juan Jiménez-Moleón, José María Huerta, Silvia de Sanjosé, Rosa del Campo, Manolis Kogevinas, Marina Pollán, Michael Pawlita, Tim Waterboer, Annemarie Boleij, Nuria Aragonés

Supplementary table S1: MCC-Spain study population characteristics

	Controls	Cases
Overall, n (%)	576 (100)	576 (100)
Age [years], mean (range)	64 (35-84)	64 (31-85)
Sex, n (%)		
Males	286 (50)	285 (50)
Females	290 (50)	291 (50)
Education, n (%)		
Low	313 (54)	371 (64)
Intermediate	156 (27)	136 (24)
High	107 (18)	69 (12)
Smoking status, n (%)		
Never	293 (51)	258 (45)
Former	166 (29)	209 (37)
Current	113 (19)	105 (18)
Missing	4	4
BMI [kg/m^2], n (%)		
<25	161 (28)	165 (29)
25- <30	274 (48)	247 (43)
≥30	141 (24)	164 (28)
Family history of CRC, n (%)		
No	518 (89)	476 (82)
Yes	58 (10)	100 (17)

BMI: Body mass index; CRC = colorectal cancer

Supplementary table S2: Association of antibody response to FliC with adenoma/CRC in the Nijmegen/Detroit studies, separately by study

Nijmegen					
FliC	Total n (%)	Controls n (%)	Cases[1] n (%)	OR (95%CI)[2]	p_{trend}
Q1	21 (28)	7 (26)	14 (28)	ref	
Q2	12 (16)	7 (26)	5 (16)	0.36 (0.08-1.54)	
Q3	15 (20)	6 (22)	9 (20)	0.75 (0.19-2.97)	
Q4	28 (37)	7 (26)	21 (37)	1.50 (0.43-5.22)	0.242
**Detroit**					
FliC	Total n (%)	Controls n (%)	Cases[1] n (%)	OR (95%CI)[2]	p_{trend}
Q1	17 (19)	12 (26)	5 (11)	ref	
Q2	17 (19)	12 (26)	5 (21)	1.00 (0.23-4.37)	
Q3	22 (24)	11 (23)	11 (25)	2.40 (0.63-9.14)	
Q4	35 (38)	12 (26)	23 (52)	**4.60 (1.31-16.14)**	**0.025**

[1]Cases include n=12 adenoma and n=37 CRC cases for Nijmegen; and n=11 adenoma and n=33 CRC cases in the Detroit study; [2]Logistic regression model without adjustments; Age and sex were available but only for part of the study; significant associations (p-value < 0.05) are marked in bold font

Supplementary table S3: Antibody response to FliC and association with SGG Gallo2178 and Gallo2179 antibody positivity in the Nijmegen/Detroit studies, separately by study

Nijmegen

	Gallo2178			Gallo2179		
FliC quartile	neg	pos	p-value[2]	neg	pos	p-value[2]
**Total**	(n=70)	(n=6)		(n=68)	(n=8)	
Q1	19 (27)	2 (33)		18 (27)	3 (38)	
Q2	12 (17)	0 (0)		12 (18)	0 (0)	
Q3	12 (17)	3 (50)		10 (15)	5 (63)	
Q4	27 (39)	1 (17)	0.183	28 (41)	0 (0)	**0.004**
**Controls**	(n=23)	(n=4)		(n=22)	(n=5)	
Q1	5 (22)	2 (50)		4 (18)	3 (60)	
Q2	7 (30)	0 (0)		7 (32)	0 (0)	
Q3	5 (22)	1 (25)		4 (18)	2 (40)	
Q4	6 (26)	1 (25)	0.515	7 (32)	0 (0)	0.078
**Cases[1]**	(n=47)	(n=2)		(n=46)	(n=3)	
Q1	14 (30)	0 (0)		14 (30)	0 (0)	
Q2	5 (11)	0 (0)		5 (11)	0 (0)	
Q3	7 (15)	2 (100)		6 (13)	3 (100)	
Q4	21 (45)	0 (0)	**0.026**	21 (46)	0 (0)	**0.003**

Detroit

	Gallo2178			Gallo2179		
FliC quartile	neg	pos	p-value[2]	neg	pos	p-value[2]
**Total**	(n=80)	(n=11)		(n=83)	(n=8)	
Q1	17 (21)	0 (0)		17 (21)	0 (0)	
Q2	15 (19)	2 (18)		16 (19)	1 (13)	
Q3	17 (21)	5 (46)		19 (23)	3 (38)	
Q4	31 (39)	4 (36)	0.195	31 (37)	4 (50)	0.429
**Controls**	(n=42)	(n=5)		(n=44)	(n=3)	
Q1	12 (29)	0 (0)		12 (27)	0 (0)	
Q2	11 (26)	1 (20)		11 (25)	1 (33)	
Q3	9 (21)	2 (40)		10 (23)	1 (33)	
Q4	10 (24)	2 (40)	0.455	11 (25)	1 (33)	0.776
**Cases[1]**	(n=38)	(n=6)		(n=39)	(n=5)	
Q1	5 (13)	0 (0)		5 (13)	0 (0)	
Q2	4 (11)	1 (17)		5 (13)	0 (0)	
Q3	8 (21)	3 (50)		9 (23)	2 (40)	
Q4	21 (55)	2 (33)	0.365	20 (51)	3 (60)	0.603

[1]Cases include n=12 adenoma and n=37 CRC cases for Nijmegen; and n=11 adenoma and n=33 CRC cases in the Detroit study; [2]Chi-square test; significant associations (p-value < 0.05) are marked in bold font
